# A metabolic entry point into micro-chlorophagy: insights from impaired maltose metabolism

**DOI:** 10.1080/27694127.2026.2721027

**Published:** 2026-08-26

**Authors:** Sakuya Nakamura, Shinya Hagihara, Masanori Izumi

**Affiliations:** RIKEN Center for Sustainable Resource Science (CSRS), Wako, Japan

**Keywords:** *Arabidopsis*, autophagy, chlorophagy, chloroplast, maltose metabolism, plant

## Abstract

As the site of photosynthesis and carbohydrate metabolism, chloroplasts are essential for phototrophic plant growth. The selective elimination of damaged chloroplasts by autophagy, a process known as chlorophagy, is crucial for chloroplast quality control. However, the mechanisms that activate chlorophagy and that underline its specificity remain poorly understood. Our recent study identified changes in maltose metabolism as a previously unrecognized trigger of a micro-chlorophagy program that depends on components of the core autophagy machinery. These findings provide a new starting point for uncovering the molecular principles that govern micro-chlorophagy. Here, we discuss emerging evidence showing that metabolic changes induce autophagic chloroplast turnover and outline the key challenges in defining the signals that initiate micro-chlorophagy.

Autophagy mediates the turnover of chloroplasts through a selective process known as chlorophagy. Chloroplasts are the sites of photosynthesis, where light energy is converted into chemical energy and atmospheric carbon dioxide is assimilated into carbohydrates. When chloroplasts are exposed to excess light that exceeds the capacity of the photosynthetic machinery; however, reactive oxygen species accumulate and cause photodamage. We previously identified a micro-chlorophagy pathway in *Arabidopsis thaliana* leaves that selectively removes chloroplasts damaged by transient high-light stress. This process requires several core autophagy-related (ATG) proteins and chloroplasts are engulfed directly by the vacuolar membrane. As with other forms of selective micro-autophagy, this pathway is expected to involve mechanisms that couple chloroplast damage with chlorophagy activation, thus allowing cells to distinguish damaged chloroplasts from healthy ones. However, the signals that mark individual chloroplasts and initiate micro-chlorophagy remain largely unknown.

We explored micro-chlorophagy-inducing conditions beyond photodamage to gain insight into the signals that activate this pathway. In our recent study [1], we focused on disruption of chloroplast carbohydrate metabolism caused by the loss of the chloroplast inner envelope membrane-localized maltose exporter MALTOSE EXCESS 1 (MEX1). The major findings of this study are resumed in Figure 1. In wild-type plants, chloroplasts store photosynthetically derived carbon as starch during the day and degrade it at night, releasing maltose for export into the cytosol via MEX1. In mature leaves of the *mex1* mutant, maltose accumulated to levels more than 70-fold higher than in wild-type plants, resulting in severe chlorosis and striking chloroplast abnormalities, including swelling and globular membrane protrusions. Chloroplasts undergoing degradation accumulated in the vacuolar lumen in *mex1* leaves. Their vacuolar accumulation was dependent on the core autophagy machinery, requiring ATG7 and ATG10, and was accompanied by ATG8 accumulation on the surface of swollen chloroplasts. Together, these observations establish defective maltose metabolism as a previously unrecognized trigger of a micro-chlorophagy that depends on at least part of the ATG machinery, specifically the two-ubiquitin-like conjugation systems.

**Figure 1.**
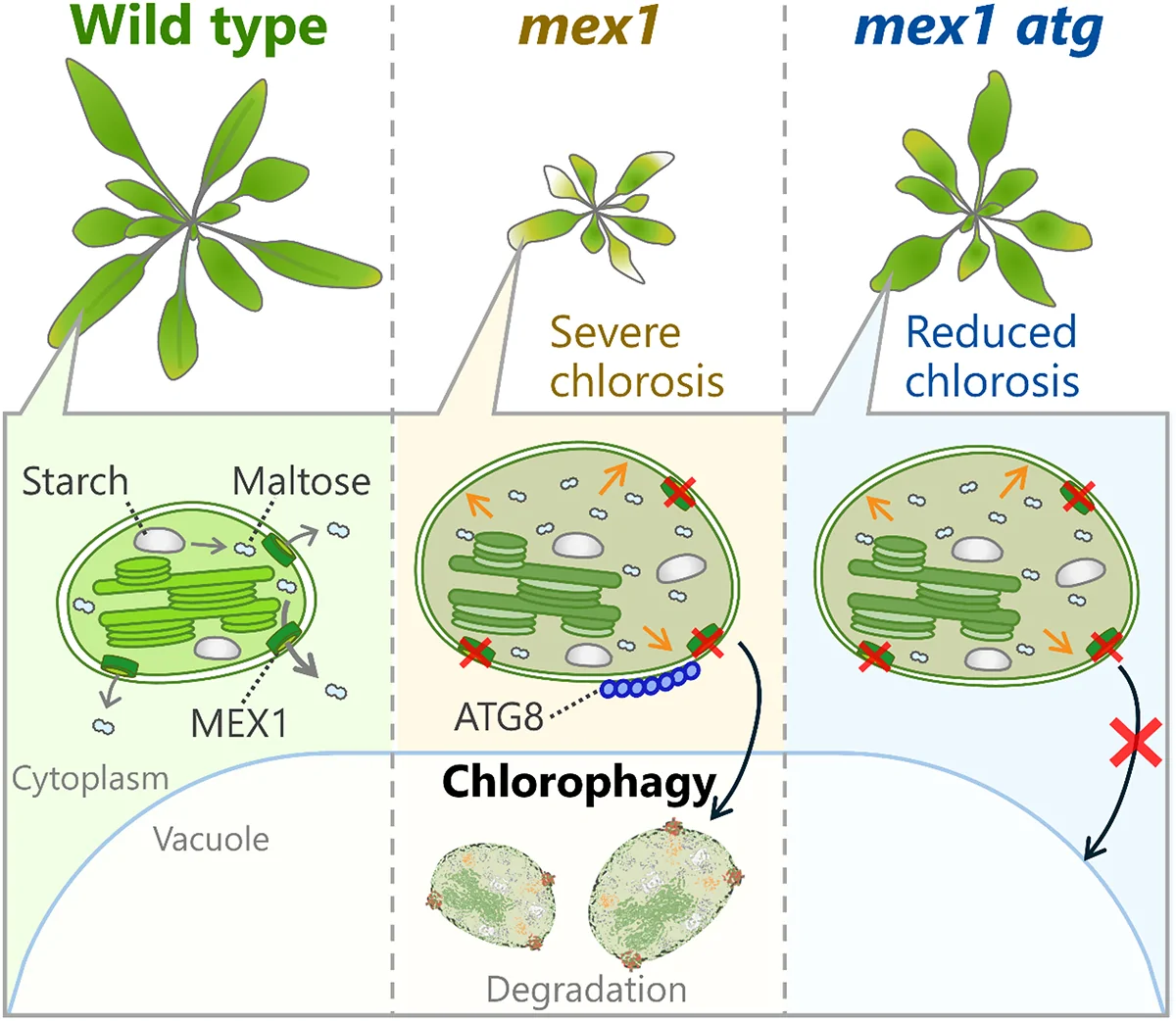
A model of micro-chlorophagy triggered by maltose hyperaccumulation and the associated plant phenotypes in *Arabidopsis*. During the night, in wild-type plants, starch degradation in chloroplasts releases maltose, which is exported to the cytosol by the chloroplast inner envelope membrane transporter MEX1. Loss of MEX1 function (*mex1*) blocks maltose export, leading to excessive maltose accumulation within chloroplasts. This metabolic defect is associated with leaf chlorosis and abnormal chloroplast morphology, including swelling and globular envelope protrusions. These chloroplast abnormalities are proposed to induce micro-chlorophagy, resulting in vacuolar chloroplast degradation. Micro-chlorophagy in *mex1* leaves depends on at least part of the core components of the autophagy machinery and is abolished in *mex1 atg7* or *mex1 atg10* double mutant plants (*mex1 atg*). Disruption of micro-chlorophagy in *mex1 atg* double mutant plants partially alleviates chlorosis and growth retardation in *mex1* plants, suggesting that excessive micro-chlorophagy contributes to the phenotypic consequences of maltose hyperaccumulation.

Chlorophagy is rarely observed in young developing leaves of the *mex1* mutant, but becomes progressively enhanced as leaves mature, coinciding with the worsening of chlorotic phenotypes. This temporal pattern is likely linked to the progressive accumulation of maltose within *mex1* chloroplasts, which occurs through repeated day-and-night cycles of starch synthesis and degradation. This observation supports the notion that chlorophagy is not triggered simply by the loss of MEX1 itself, but rather when the resulting metabolic and structural consequences exceed a critical threshold. Once chloroplast swelling or other downstream stress-associated changes reach this threshold, affected chloroplasts may acquire signals, as for example mitochondria, that recruit the machinery that leads to their local engulfment by vacuoles. Identifying the nature of these signals will be important for understanding how chlorophagy is initiated.

Interestingly, the *mex1 atg7* and *mex1 atg10* double mutants exhibited improved growth and reduced leaf chlorosis compared with the *mex1* single mutants. This genetic suppression suggests that micro-chlorophagy activation in *mex1* leaves contributes to the severity of phenotypes associated with maltose hyperaccumulation. The expression of several core *ATG* genes was elevated in *mex1* leaves, indicating that maltose hyperaccumulation stimulates the expression of the autophagy machinery. Together, these results suggest that micro-chlorophagy is overactivated in *mex1* leaves. To gain insight into the signaling pathways underlying these responses, we compared transcriptomic changes in *mex1* leaves with those induced by high-light stress, a distinct trigger of chlorophagy. RNA-sequencing analysis revealed a shared transcriptional signature characterized by the activation of genes associated with pathogen defense, oxidative stress, and wound responses. Such transcriptional changes are closely linked to signaling pathways mediated by the plant hormones salicylic acid and jasmonic acid. This shared transcriptional signature suggests that these pathways may participate in the cellular response to chloroplast damage, a key trigger of chlorophagy induction.

Our findings raise the possibility that defects in chloroplast metabolism or metabolite transport generate a chloroplast damage that triggers chlorophagy under a broader range of environmental and developmental conditions. Because high-light treatment also generates a subpopulation of chloroplasts that exhibit swelling and ATG8 association, features reminiscent of those observed in *mex1* leaves, photodamage might disrupt metabolic processes such as maltose metabolism in individual chloroplasts, thereby contributing to their selective degradation. However, an important distinction exists between these two conditions. In *mex1* leaves, the metabolic defect is present in all chloroplasts, whereas high light-induced swelling occurs only in a small subset of the chloroplast population. Consequently, metabolic alterations occurring specifically in chloroplasts undergoing micro-chlorophagy in high light-treated leaves are likely masked when metabolite profiling is performed at the whole-leaf level. Indeed, high light-treated leaves exhibited only a modest increase in maltose content, far smaller than that observed in *mex1* leaves [1]. Directly testing whether metabolic dysfunction in individual chloroplasts triggers micro-chlorophagy will require approaches capable of resolving metabolic states at the level of single organelles. Alternatively, metabolic dysfunction and photodamage may converge on a common chloroplast damage signal that marks chloroplasts for recognition by the micro-chlorophagy machinery.

Taken together, our recent study established *mex1* plants as a valuable model for elucidating the mechanisms of chlorophagy and provided important insights into the signals that trigger this process. Future studies should focus on identifying the threshold signals that mark chloroplasts for degradation, clarifying how chlorophagy interacts with hormone-mediated signaling pathways, and determining the metabolic changes that occur specifically in chloroplasts destined for chlorophagic degradation.

## Data Availability

Data sharing is not applicable to this article as no data were created or analyzed in this study.

## Abbreviations

ATG: autophagy related
MEX1: maltose excess 1
